# Excess of Aminopeptidase A in the Brain Elevates Blood Pressure via the Angiotensin II Type 1 and Bradykinin B2 Receptors without Dipsogenic Effect

**DOI:** 10.1155/2017/3967595

**Published:** 2017-03-22

**Authors:** Takuto Nakamura, Masanobu Yamazato, Akio Ishida, Yusuke Ohya

**Affiliations:** Department of Cardiovascular Medicine, Nephrology and Neurology, Graduate School of Medicine, University of the Ryukyus, Okinawa Prefecture, Japan

## Abstract

Aminopeptidase A (APA) cleaves angiotensin (Ang) II, kallidin, and other related peptides. In the brain, it activates the renin angiotensin system and causes hypertension. Limited data are available on the dipsogenic effect of APA and pressor effect of degraded peptides of APA such as bradykinin. Wistar-Kyoto rats received intracerebroventricular (icv) APA in a conscious, unrestrained state after pretreatment with (i) vehicle, (ii) 80 *μ*g of telmisartan, an Ang II type-1 (AT1) receptor blocker, (iii) 800 nmol of amastatin, an aminopeptidase inhibitor, and (iv) 1 nmol of HOE-140, a bradykinin B2 receptor blocker. Icv administration of 400 and 800 ng of APA increased blood pressure by 12.6 ± 3.0 and 19.0 ± 3.1 mmHg, respectively. APA did not evoke drinking behavior. Pressor response to APA was attenuated on pretreatment with telmisartan (vehicle: 22.1 ± 2.2 mmHg versus telmisartan: 10.4 ± 3.2 mmHg). Pressor response to APA was also attenuated with amastatin and HOE-140 (vehicle: 26.5 ± 1.1 mmHg, amastatin: 14.4 ± 4.2 mmHg, HOE-140: 16.4 ± 2.2 mmHg). In conclusion, APA increase in the brain evokes a pressor response via enzymatic activity without dipsogenic effect. AT1 receptors and B2 receptors in the brain may contribute to the APA-induced pressor response.

## 1. Introduction

Aminopeptidase A (APA) is an enzyme that hydrolyzes the N-terminal amino acid of peptides and expressed in both periphery and brain. Peripheral APA was reported to have potency to act as both protective [1, 2] and harmful [3, 4] agent on cardiovascular disease. Several studies reported that brain APA contributes to neurogenic hypertension. Brain APA activity was higher in spontaneously hypertensive rats (SHR) than in Wistar-Kyoto (WKY) rats [5]. Moreover, brain APA activity in SHR increased with the development of hypertension [6]. Intracerebroventricular (icv) administration of EC33, the specific APA activity inhibitor, decreased blood pressure in SHR [7]. Orally administered RB150, which is a prodrug of EC33, also decreased blood pressure in DOCA-salt rats [8] and SHR [9]. RB150 can cross the blood-brain barrier and gets degraded to EC33 in the brain; oral administration of RB150 inhibits brain APA activity [8]. This evidence suggests that increased brain APA activity participates in the maintenance of hypertension.

Research regarding the role of APA in the brain has focused on degradation of angiotensin (Ang) II to Ang III. Icv administration of EC33 and RB150 inhibited the degradation of Ang II to Ang III in the brains of Swiss mice [10], while that of EC33 abolished the pressor response of icv-administered Ang II. In contrast, icv administration of EC33 did not change the pressor response of icv-administered Ang III [7]. Therefore, conversion of Ang II to Ang III by APA may require elevated blood pressure in the brain [11–13]. However, little is known of the dipsogenic effect of APA. Icv administration of both Ang II and Ang III evokes pressor and dipsogenic responses via the Ang II type-1 (AT1) receptor [14], and Faber et al. reported that APA knock-out mice increase their water intake [15]. Therefore, systemic APA may participate in dipsogenic regulation. However, the dipsogenic effect of brain APA remains unclear.

Furthermore, the cardiovascular effect of brain APA activity has not been resolved beyond the degradation of Ang II to Ang III. It is important to note that APA is not specific to Ang II. Goto et al. reported that neurokinin B, cholecystokinin, and chromogranin A were efficiently cleaved by APA, and kallidin could also be cleaved by APA [16]. A role for the degradation of these peptides in cardiovascular regulation is unresolved. In the present study, we focused on kallidin, a precursor peptide of bradykinin. Bradykinin in the cerebrospinal fluid (CSF) [17] and bradykinin B1 and B2 receptors [18] increased in SHR. The pressor response evoked by icv-administered bradykinin was greater in SHR than in WKY [19] and increased with aging in SHR [18]. Both bradykinin B1 [20, 21] and B2 receptor were reported to contribute neurogenic hypertension; however, B2 receptor is constitutively expressed in physiological condition, whereas B1 receptor is induced by injury or inflammatory conditions [22]. Icv administration of the B2 receptor blocker HOE-140 blocked the pressor response of icv-administered bradykinin in WKY rats and SHR [18]. Microinjection of HOE-140 into the rostral ventrolateral medulla decreased blood pressure to a greater extent in SHR than in WKY rats [19, 23]. These results suggest that bradykinin in the brain plays a role in maintaining the blood pressure of hypertensive rats.

We anticipated that APA shifts the balance between substrates and degraded peptides via enzymatic activity to regulate cardiovascular control. We hypothesized that increased brain APA activity would evoke a pressor response and drinking behavior via AT1 receptor stimulation, and a decrease in kallidin and increase in bradykinin as a consequence of APA activity would increase B2 receptor contribution to the pressor response. To evaluate the role of excess brain APA, we measured the duration of drinking time after icv administration of APA and the pressor response evoked by APA under AT1 receptor or B2 receptor blockade.

## 2. Materials and Methods

### 2.1. Animals

Eleven- to 14-week-old male WKY/Izm rats were used for the experiments. WKY/Izm were provided from the Disease Model Cooperative Research Association, Kyoto, Japan, and were placed under artificial light (12 h light/12 h dark cycle) with a normal standard diet (CE-2; Japan CLEA) and water provided ad libitum. All procedures were in accordance with the National Institutes of Health Guidelines for the Care and Use of Laboratory Animals. The protocol was reviewed and approved by the Animal Care and Use Committee, University of the Ryukyus.

### 2.2. Histological Examination

Fixation and immunostaining procedures used in this study were previously described [24]. In brief, the rats were transcardially perfused with 70 ml of cold sodium phosphate buffered saline (PBS: 0.01 M phosphate buffer containing 0.9% sodium chloride, pH 7.2, 4°C) followed by 35 ml of 4% paraformaldehyde in PBS. The brain stem was removed, postfixed in 4% paraformaldehyde solution for 1 h, and transferred to a PBS containing 20% sucrose (pH 7.4). Frozen brain tissues were sectioned in the coronal plane (5 *μ*m). Sections were first incubated with 0.3% H_2_O_2_ in methanol for 30 min, followed by incubation with 5% skim milk in tris-buffered saline (20 mM tris-HCl, 0.9% sodium chloride, 0.02% tween 20, pH 7.4) for 60 min. They were incubated with goat polyclonal anti-APA antibody (1 : 100, ab36122, abcam) for 120 min, biotinylated rabbit anti-goat immunoglobulin G for 120 min, and avidin-biotin-peroxidase complex reagents for 30 min and finally stained with diaminobenzidine solution for 8 min according to the manufacturer's instructions (Vector Laboratories). Each step was followed by washing the sections with PBS. Sections incubated without primary antibodies were used as negative controls.

### 2.3. Drugs

Telmisartan was kindly gifted by Boehringer Ingelheim. It was dissolved in dimethyl sulfoxide (DMSO). HOE-140 (Sigma-Aldrich Japan), amastatin (Santa Cruz Biotechnology), Ang II (Sigma-Aldrich Japan), and Ang III (Sigma-Aldrich Japan) were dissolved in artificial CSF (aCSF, 133.3 mM sodium chloride, 3.4 mM potassium chloride, 1.3 mM calcium chloride, 1.2 mM magnesium chloride, 0.6 mM sodium dihydrogen orthophosphate, 32.0 mM sodium bicarbonate, and 3.4 mM glucose) [15]. Mouse recombinant APA (Sino Biological) compound contains 1.28 mmol sodium chloride per 1 mg of APA. APA was dissolved and electrolyte concentration adjusted to match aCSF. For example, APA was dissolved to 0.1 mg/ml in a vehicle (5 mM sodium chloride, 3.4 mM potassium chloride, 1.3 mM calcium chloride, 1.2 mM magnesium chloride, 0.6 mM sodium dihydrogen orthophosphate, 32.0 mM sodium bicarbonate, and 3.4 mM glucose).

### 2.4. Surgical Procedure

#### 2.4.1. Implantation of Arterial Catheter for Blood Pressure Measurement

Rats were anesthetized with an intraperitoneal injection of 50 mg/kg of pentobarbital sodium, and vascular catheters were inserted through the right femoral artery for blood pressure measurement. Catheters were exteriorized at the interscapular region through a subcutaneous tunnel.

#### 2.4.2. Implantation of Guide Cannula for Icv Administration

Surgical procedures used in this study were previously described [25]. In brief, rats were placed on a stereotaxic frame (Narishige Scientific Instruments) in a prone position. A hole was drilled through the dorsal surface of the cranium 1.0 mm posterior to the bregma and 1.8 mm lateral to the midline. A 21-gauge stainless steel guide cannula was lowered 3 mm vertically from the skull surface toward the lateral ventricle. The cannula was anchored to the skull using cyanoacrylate. Before and after the surgery, each rat received an intramuscular injection of 10,000 U of penicillin G for prophylaxis.

### 2.5. Experiment Protocol

At least 2 days after arterial catheter implantation and icv cannula, the rats were placed in a plastic cage and freely allowed to move. The arterial catheter was connected to a pressure transducer, and arterial pressure and heart rate were recorded using the powerlab system (AD Instrument) in a conscious, unrestrained state. Drugs were icv-administered by inserting a 27-gauge stainless steel needle into the guide cannula so that it extended 2 mm beyond the tip of the guide into the lateral ventricle.

*Protocol 1 (Effect of Icv Administration of APA).* Rats received icv administration of vehicle (*n* = 4), 400 ng/8 *μ*l of APA (*n* = 5), or 800 ng/8 *μ*l APA (*n* = 4). They were allowed free access to water. We recorded blood pressure, heart rate, and drinking time after icv administration of APA in a conscious, unrestrained state. Furthermore, a different set of rats received icv administration of 25 ng/2 *μ*l of Ang II (*n* = 4) and 25 ng/2 *μ*l of Ang III (*n* = 4) to establish a positive control for drinking time. This dose of Ang II and Ang III evoked similar pressor responses to icv-administered 800 ng of APA.

*Protocol 2 (Icv Administration of APA under AT1 Receptor Blockade, Inhibition of Aminopeptidase Activity, or Bradykinin B2 Receptor Blockade)*. Rats received icv administration of DMSO as a vehicle (*n* = 5) or 80 *μ*g/4 *μ*l telmisartan (*n* = 5). This dose of telmisartan completely blocked the pressor response and drinking behavior induced by 25 ng of Ang II for at least 2 h. At least 30 min after the administration of telmisartan, rats received 800 ng/8 *μ*l of APA. Finally, rats received 25 ng of Ang II to confirm AT1 receptor blockade. Furthermore, different groups of rats received icv administration of aCSF (*n* = 6), 800 nmol/8 *μ*l of amastatin (*n* = 6), or 1 nmol/8 *μ*l of HOE-140 (*n* = 7). The dose of amastatin was expected to block 97% of the activity of 800 ng of APA based on our preliminary in vitro experiment and the dose of HOE-140 was reported to inhibit the pressor response to icv administration of an equimolar dose of bradykinin [18]. At least 30 min after pretreatment with the drugs, rats received 800 ng/8 *μ*l of APA.

At least 30 min after icv administration of APA, we administered 25 ng of Ang II to confirm the correct placement of icv cannula and the absence of nonspecific inhibition of pretreatment drugs. All icv administrations were performed over a period of 20 s. At the end of each experiment, the correct placement of the icv cannula was rechecked with postmortem icv administration of methylene blue.

### 2.6. Data and Statistical Analysis

Time dependent change and maximal change of mean arterial pressure (MAP) after icv administration of drugs were measured for each rat. Results were analyzed using ANOVA or Student's *t*-tests. Values are expressed as mean ± SE. A *p* value of < 0.05 was considered to be significant.

## 3. Results

### 3.1. Histological Examination

**Figure 1:**
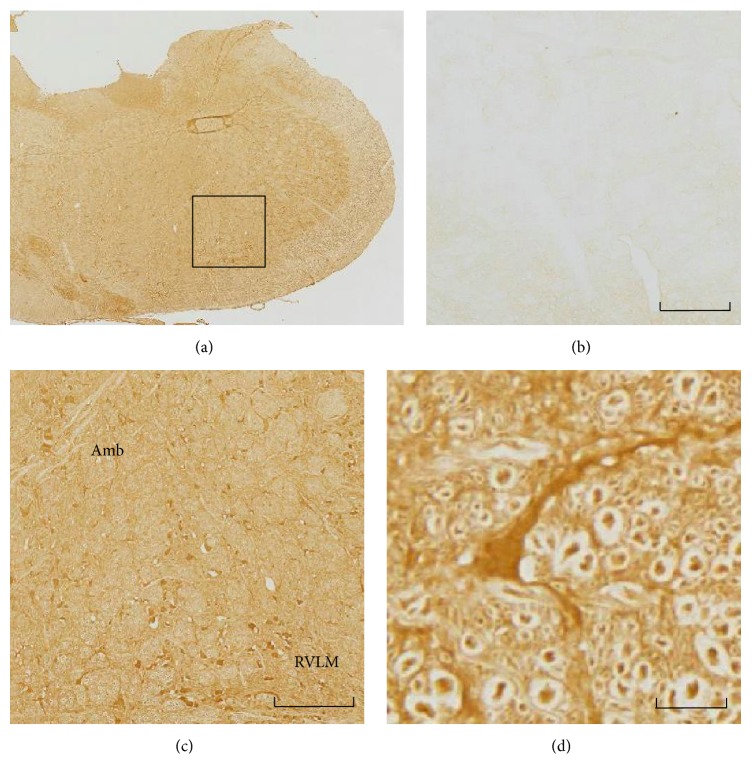
Immunoreactivity of the aminopeptidase A (APA) in the medulla oblongata of Wistar-Kyoto rats. Sections were immunostained with anti-APA antibody as described in the Materials and Methods. (a) The low-magnification photograph of medulla oblongata. APA immunoreactivity is diffusely distributed. The square in the photo indicates the area shown in (b) and (c). (b) Negative control. (c) APA immunoreactivities were observed in ambiguous nucleus (Amb) and rostral ventrolateral medulla (RVLM). (d) Higher magnification of APA-stained neuron. Neurons and neuropils were positive for APA immunoreactivity. The scale bar denotes 200 *μ*m in (a) and (b) and 20 *μ*m in (c).

Figure 1 shows immunoreactivity of APA in the medulla oblongata. Immunoreactivity of APA was distributed in the rostral ventrolateral medulla and ambiguous nucleus and was observed in the cytoplasm of neurons and neuropils.

### 3.2. Effect of Icv Administration of APA

**Figure 2:**
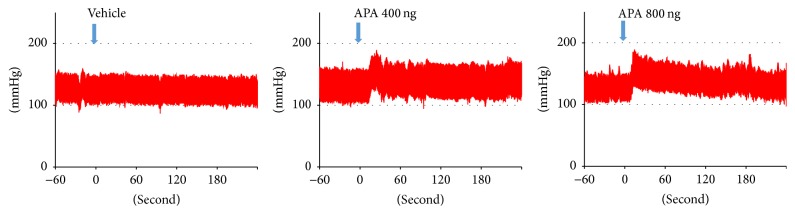
Representative traces of arterial pressure after intracerebroventricular (icv) administration of aminopeptidase A (APA). Wistar-Kyoto rats received icv administration of vehicle or APA in a conscious, unrestrained state. Icv of APA rapidly increased blood pressure. Then, blood pressure gradually decreased. The duration of pressor response was prolonged in a dose-dependent manner. Arrow: time for drug administration.

To evaluate cardiovascular and dipsogenic effects of brain APA, we performed icv administration of APA.  Figure 2 shows representative traces of arterial pressure after icv administration of APA. Icv administration of APA rapidly increased arterial pressure (Table 1). The duration of pressor response persisted in a dose-dependent manner.

**Figure 3:**
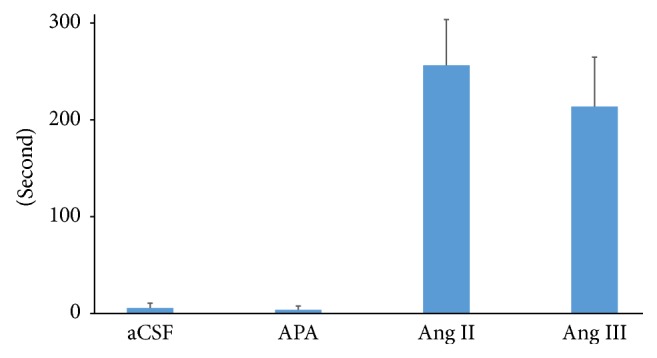
Duration of drinking time after intracerebroventricular (icv) administration of angiotensin (Ang) II, Ang III, or aminopeptidase A (APA). Duration of drinking time was measured after icv administration of aCSF, 25 ng of Ang II, 25 ng of Ang III, or 800 ng of APA. This dose of Ang II or Ang III evoked similar pressor response to 800 ng of APA. After icv administration of CSF, drinking behavior was not observed; after icv administration of Ang II and Ang III, continuous drinking behavior was observed; after icv administration of APA, drinking behavior was not observed. Values are expressed as mean ± SE.

Icv administration of Ang II or Ang III immediately evoked a behavior of searching for a water bottle. After finding the bottle, the rats started drinking water. A continuous drinking behavior was observed, and the drinking time durations evoked by Ang II and Ang III were 256 ± 47 and 214 ± 51 s, respectively. Icv administration of Ang II and Ang III increased blood pressure by 17.8 ± 4.5 and 16.4 ± 3.3 mmHg, respectively. Both drinking time and pressor response were not different between icv-administered Ang II and Ang III. The drinking behavior and pressor response were abolished by icv administration of 80 *μ*g of telmisartan. Contrary to icv administration of Ang II or Ang III, icv administration of APA did not evoke drinking behavior (Figure 3).

### 3.3. Icv Administration of APA under AT1 Receptor Blockade, Inhibition of Aminopeptidase Activity, or B2 Receptor Blockade

**Figure 4:**
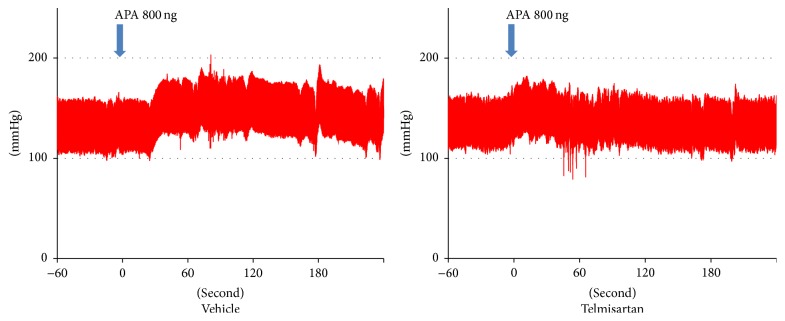
Representative traces of arterial pressure after intracerebroventricular (icv) administration of aminopeptidase A (APA) following pretreatment with telmisartan. Thirty minutes prior to the icv administration of 800 ng of APA, Wistar-Kyoto rats received icv administration of vehicle (*n* = 5) or 80 *μ*g of telmisartan (*n* = 5) in a conscious, unrestrained state. Icv administration of telmisartan or vehicle did not change the baseline MAP. Pretreatment with telmisartan significantly attenuated the pressor response of APA. Arrow: time for APA administration.

Figure 4 shows representative traces of arterial pressure after icv administration of APA following pretreatment with vehicle or telmisartan. Icv administration of 80 *μ*g/4 *μ*l of telmisartan or 4 *μ*l of vehicle did not change the baseline arterial pressure (Table 2). Icv administration of telmisartan significantly attenuated the pressor response of APA (Figure 4, Table 2). The time dependent change of the pressor response to APA was not different with icv treatment (Table 2).

**Figure 5:**
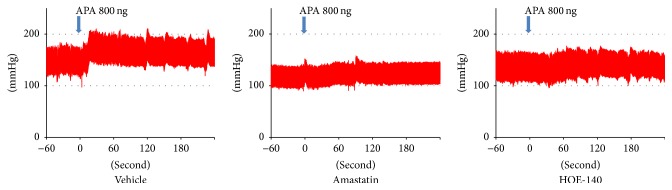
Representative traces of arterial pressure after intracerebroventricular (icv) administration of aminopeptidase A (APA) following pretreatment with amastatin or HOE-140. Thirty minutes prior to the icv administration of 800 ng of APA, Wistar-Kyoto rats received icv administration of the following drugs: vehicle (*n* = 6), 800 nmol of amastatin (*n* = 6), or 1 nmol of HOE-140 (*n* = 7). Icv administration of vehicle, amastatin, and HOE-140 did not change the baseline MAP. Pretreatment of amastatin and HOE-140 significantly attenuated the pressor response to APA. Arrow: time for APA administration.

Figure 5 shows representative traces of arterial pressure of icv-administered APA following pretreatment with amastatin. Icv administration of 800 nmol of amastatin did not change the baseline blood pressure (Table 3). Icv administration of amastatin significantly attenuated the pressor response of APA (Figure 5, Table 3).

Figure 5 shows representative traces of arterial pressure for icv-administered APA following pretreatment with HOE-140. Icv administration of 1 nmol of HOE-140 did not change the baseline blood pressure (Table 3). Icv administration of HOE-140 significantly attenuated the pressor response to APA (Figure 5, Table 3). Finally, administration of Ang II increased blood pressure by 31.9 ± 3.6 mmHg in pretreatment with aCSF, 32.3 ± 5.3 mmHg in pretreatment with amastatin, and 22.2 ± 4.7 mmHg in pretreatment with HOE-140. Magnitudes of pressor response were not significantly different between each group.

Furthermore, we performed icv administration of APA following icv coinjection of 80 *μ*g of telmisartan and 1 nmol of HOE-140. Icv administration of APA after coinjection of telmisartan and HOE-140 increased blood pressure by 13.8 ± 2.6 mmHg (*n* = 3). There seems to be no additive effect of B2 receptor blockade to AT1 receptor blockade.

**Figure 6:**
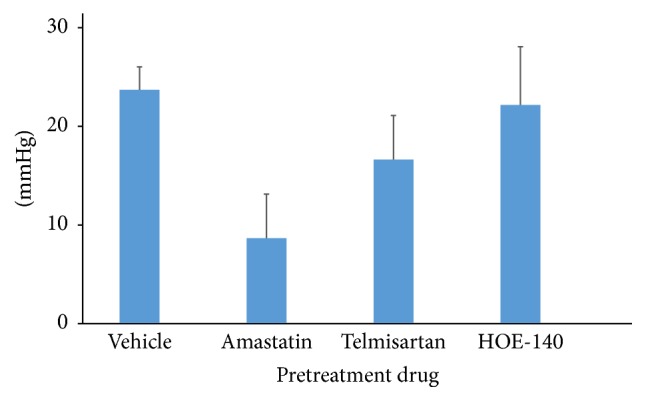
The pressor response of lower dose of aminopeptidase A (APA). Wistar-Kyoto rats received icv administration of 200 ng of APA following treatment of aCSF (*n* = 4) or each blocker. The pressor response of lower dose of APA seemed to be smaller in 800 nmol of amastatin (*n* = 3) than in 80 *μ*g of telmisartan (*n* = 2) or 1 nmol of HOE-140 (*n* = 2).

Finally, we performed additional experiment using lower dose of APA (200 ng) under inhibition of aminopeptidase activity, AT1 receptor blockade, or B2 receptor blockade using same dose of the blockers as protocol 2. Icv administration of 200 ng of APA increased blood pressure by 23.6 ± 2.3 mmHg in pretreatment with aCSF (*n* = 4). Pressor response of APA seemed to be smaller in amastatin (8.6 ± 4.5 mmHg, *n* = 3) than in telmisartan (16.5 ± 4.5 mmHg, *n* = 2) or HOE-140 (22.0 ± 5.9 mmHg, *n* = 2) (Figure 6). Two hundreds ng of APA did not induce drinking behavior (6.3 ± 6.3 s in pretreatment with aCSF, 5 ± 5 s in pretreatment with telmisartan).

## 4. Discussion

This study shows that (i) increase in brain APA elevates blood pressure via enzymatic activity in a dose-dependent manner, (ii) increase in brain APA did not evoke drinking behavior, and (iii) AT1 and B2 receptors contribute to the pressor response to brain APA. These results suggested that increased brain APA activity evokes a pressor response via the AT1 and B2 receptors without dipsogenic effect.

APA activity is diffusely distributed in the brain of rats [5]. Brain APA activity increased in SHR [5, 6]. In humans, APA activity and immunoreactivity are also diffusely distributed in the brain including the cardiovascular regulatory nuclei [26]. We also observed APA immunoreactivity diffusely in the medulla oblongata including cardiovascular regulatory areas. Our study intended to evaluate a role for brain APA in cardiovascular regulation.

Wright et al. showed that icv administration of placenta-derived APA increased blood pressure, and the pressor response was attenuated by icv administration of AT1 receptor blocker (ARB) under anesthesia in WKY rats and SHR [27]. In the present study, we supported their observation using conscious rats and recombinant APA. In the present study, inhibition of APA activity or AT1 receptor blockade attenuated the pressor response to icv-administered APA. As previously reported, inhibition of APA activity decreases blood pressure in hypertensive animals [7–9]. Therefore, brain APA is believed to act as a pressor agent via its enzymatic activity through the AT1 receptor.

Brain AT1 receptor stimulation induces blood pressure elevation and drinking behavior. However, present study showed that brain APA increased blood pressure elevation via the AT1 receptor, in contrast, did not induce drinking behavior. This discrepancy has not been fully understood. One possible explanation is that Ang II and Ang III have similar potent dipsogen; therefore, degradation of Ang II to Ang III did not evoke drinking behavior. Another possible explanation is that the response of icv-administered APA was sum of heterogeneous reactions of endogenous substrate and metabolites affecting many different cardiovascular regulatory areas in the brain simultaneously. This complex effect might be causing the difference in drinking behavior.

In the present study, amastatin had more potent effect than ARB on attenuation of the pressor response of APA. This result may suggest that the AT1 receptor mediated pressor response is a part of the pressor response of APA. In the present study, pressor response to APA was attenuated by not only ARB but also B2 receptor blocker. Excess APA is expected to convert kallidin to bradykinin even in the presence of Ca^2+^ which attenuates this process [16], such condition occurs in CSF. Both kallidin and bradykinin have a pressor potency in the brain. Bradykinin acts as a pressor peptide with longer duration than kallidin [28]. Therefore, degradation of kallidin to bradykinin is expected to increase blood pressure via prolonged pressor action and to function in the maintenance of hypertension. Further studies are needed to clarify the role of B2 receptor in the condition of increased APA.

In summary, increased brain APA may participate in the maintenance of hypertension. Brain APA is believed to modulate the renin-Ang system and to be a therapeutic target in hypertension and cardiac dysfunction [29]. Phase I study of a centrally acting APA inhibitor prodrug has already been performed [30]. We believe that inhibition of brain APA may be a unique therapeutic target, which affects several cardiovascular peptides in the brain as shown in the B2 receptor. In addition, APA inhibition may be superior in the environment that is prone to dehydration. Further studies are needed to clarify the role of APA and its related peptides in the brain and periphery.

## 5. Conclusion

This study shows that excessive APA in the brain elevates blood pressure via its enzymatic activity through AT1 receptor and B2 receptor without dipsogenic effect. APA in the brain may be a unique therapeutic target for neurogenic hypertension that affects multireceptor mediated blood pressure regulation.

## Figures and Tables

**Table 1:** Intracerebroventricular administration of aCSF, 400 ng of aminopeptidase A (APA), and 800 ng of APA.

	Change of MAP (mmHg)	Time to achieve peak pressor response (s)	Duration of pressor response (s)
Vehicle	0.3 ± 1.3	—	—
APA 400 ng	12.6 ± 3.0^*∗*^	29 ± 5	263 ± 81
APA 800 ng	19.0 ± 3.1^*∗*^	28 ± 17	780 ± 102^*∗∗*^

Values are expressed as mean ± SE. Results were analyzed using ANOVA. ^*∗*^*p* < 0.05 versus vehicle; ^*∗∗*^*p* < 0.05 versus 400 ng of APA.

**Table 2:** Intracerebroventricular (icv) administration of 800 ng of aminopeptidase A (APA) after icv administration of vehicle or telmisartan.

Pretreatment drug	Baseline MAP change (mmHg)	MAP change after icv of APA (mmHg)	Time to achieve peak pressor response (s)	Duration of pressor response (s)
Vehicle	1.3 ± 3.2	22.1 ± 2.2	52 ± 22	662 ± 109
Telmisartan	−2.6 ± 1.9	10.4 ± 3.2^*∗*^	41 ± 21	486 ± 153

Values are expressed as mean ± SE. Results were analyzed using Student's *t*-test. ^*∗*^*p* < 0.05 versus vehicle.

**Table 3:** Intracerebroventricular (icv) administration of 800 ng of aminopeptidase A (APA) after icv administration of vehicle, amastatin, or HOE-140.

Pretreatment drug	Baseline MAP change (mmHg)	MAP change after icv of APA (mmHg)	Time to achieve peak pressor response (s)	Duration of pressor response (s)
Vehicle	−2.8 ± 3.2	26.5 ± 1.1	20 ± 3	399 ± 62
Amastatin	−3.6 ± 3.4	14.4 ± 4.2^*∗*^	47 ± 18	618 ± 121
HOE-140	−0.3 ± 1.7	16.4 ± 2.2^*∗*^	38 ± 9	469 ± 83

Values are expressed as mean ± SE. Results were analyzed using Student's *t*-test. ^*∗*^*p* < 0.05 versus vehicle.
